# Study on the Sensing Signal Profiles for Determination of Process Window of Flexible Sensors Based on Surface Treated PDMS/CNT Composite Patches

**DOI:** 10.3390/polym10090951

**Published:** 2018-08-27

**Authors:** Joonwon Bae, Yunjung Hwang, Seon Joo Park, Ji-Hwan Ha, Hye Jun Kim, Ayeon Jang, Jaieun An, Chang-Soo Lee, Sung-Hoon Park

**Affiliations:** 1 Department of Applied Chemistry, Dongduk Women’s University, Seoul 02748, Korea; joonwonbae@gmail.com (J.B.); nari1758@kribb.re.kr (Y.H.); 20160860@dongduk.ac.kr (H.J.K.); 20160879@dongduk.ac.kr (A.J.); jxeunahn9512@gmail.com (J.A.); 2 Hazards Monitoring Bionano Research Center, Korea Research Institute of Bioscience & Biotechnology (KRIBB), Daejeon 34141, Korea; seonjoopark86@kribb.re.kr; 3 Department of Mechanical Engineering, Soongsil University, Seoul 06978, Korea; jhwan618@gmail.com; 4 Nanobiotechnology (Major), University of Science & Technology, Daejeon 34141, Korea

**Keywords:** poly(dimethylsiloxane)/carbon nanotube, cyclodextrin, flexible sensor, electrical conductivity

## Abstract

In this study, analysis of sensing signal profiles was conducted focusing on the close relationship between electrical conductivity and signal intensity in surface treated poly(dimethylsiloxane)/carbon nanotube (PDMS/CNT) composite patches for the purpose of their practical application as flexible chemical sensors. The flexible PDMS/CNT composite patches were prepared from a PDMS/CNT mixture with a two-roll apparatus. It was found that the PDMS/CNT pads showed a high electrical conductivity (10^−1^ S/m) even at low CNT loading (0.6 wt %) and a contact angle range of 105–118°. The surface of the obtained PDMS/CNT composite patches was treated using a simple bio-conjugation method to incorporate beta-cyclodextrin (beta-CD) molecules onto the surface as a sensing medium, in order to detect a model compound (Methyl Paraben, MePRB). FT-IR spectra indicated that beta-cyclodextrin molecules were effectively introduced on the surface of the PDMS/CNT patches. It was shown that the sensor signal intensity was substantially dependent on the base current value, which increased with increasing CNT loading. Accordingly, the base current value was intimately associated with the electrical conductivity of the composite patches. On the other hand, the increase in current over the base current (ΔI/I_0_) obtained after the addition of the model compound was inversely proportional to the CNT content. In this way, analysis on the sensing signal profiles of the flexible chemical sensor system was conducted to determine a process window. This study is a very useful springboard for future research activities, as more profound studies are necessary to fully understand sensing signal profiles.

## 1. Introduction

The need for ultrasensitive and flexible chemical sensors has been increasing in recent times, as it is becoming important to detect ultrasmall amounts of toxic gases/chemicals, hazardous materials, pathogens, and microorganisms for human safety [1,2,3,4,5]. To this end, it is critical to obtain electrically conductive and flexible substrates, because the use of electrical signals is considered as one of the most efficient signal transduction mechanisms in sensing and flexible substrates can be applied onto irregular surfaces such as textiles and skins [6,7]. Therefore, a substantial effort has been devoted to the production of flexible and electrically conductive substrates.

Among those various appealing candidates—such as metal thin film, graphene, conductive coating, polymer composite—poly(dimethylsiloxane)/carbon nanotube (PDMS/CNT) composite materials are very popular, because PDMS and CNT have reliable physical and chemical properties such as flexibility and electrical conductivity, respectively. Consequently, numerous research activities regarding sensors using the combination of PDMS and CNT have been extensively reported [8,9]. Most of the demonstrated sensor systems are mechanical sensors, which can monitor the changes in mechanical properties such as strain or pressure [10,11,12,13,14,15,16,17,18,19,20,21,22]. However, introduction of the PDMS/CNT composites for ultrasensitive chemical sensors is still challenging, because the responses of the PDMS/CNT composites to stimuli are not adequately rapid and selective. To overcome the obstacle, several strategies have been tried, for instance, modification of composite microstructures, surface engineering, enhancement in signal transduction, and optimization of sensor geometry [3,17,19].

As an example of developing flexible chemical sensors using PDMS/CNT composites, in the previous study, the sensing of a toxic chemical based on a surface engineered PDMS/CNT composite patch was systematically investigated. When beta-CD molecules (beta-CD) were introduced as sensing media onto the surface of the flexible PDMS/CNT composite patch, sensitive and selective identification of a model toxic chemical (methyl paraben, MePRB) was achieved [3]. The model chemical was selected as one of the most popular preservatives for cosmetics and personal hygiene products.

Even if sufficient experimental data was provided in the previous report, a detailed study and discussion on the sensing signal profiles of the chemical sensor system using the flexible PDMS/CNT composite patch was lacking. Therefore, in this article, a correlation between the electrical conductivities of the PDMS/CNT composite patches depending on CNT loading and electrical signals obtained after addition of analyte solutions was monitored. In addition, a process window for efficient production of the flexible chemical sensor was systematically determined. While the characterization and analysis methods are straightforward, this study is a sound experimental demonstration of the fundamental aspects of a flexible chemical sensor using an electrically conductive substrate, which may be useful for future applications.

## 2. Experimental

### 2.1. Materials

Poly(dimethylsiloxane) (PDMS, Sylgard 184 silicone elastomer base) was purchased from Dow-Corning (Midland, MI, USA), and the multiwalled carbon nanotubes (MWCNTs) with outer diameters of 10–20 nanometer and lengths of 100–200 μm were purchased from Hanwha Nanotech, Inc. (Seoul, Korea). A silane coupling agent, (3-aminopropyl)triethoxysilane (APTS), ethyl(dimethylaminopropyl) carbodiimide (EDC), *N*-hydroxysuccinimide (NHS), and 4-chlorobutyric acid were purchased from Aldrich (Milwaukee, WI, USA), and used as received. A host molecule, beta-cyclodextrin (beta-CD), and a target molecule, methylparaben (MePRB), were also purchased from Aldrich and used without further purification.

### 2.2. Fabrication of PDMS/CNT Composite Patches

**Figure 1:**
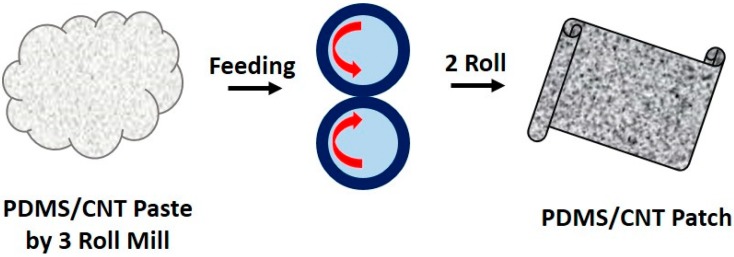
A simple schematized process for production of PDMS/CNT patches.

The process of the producing of the PDMS/CNT composite patches is summarized in Figure 1. The PDMS precursor and curing agent were mixed at a weight ratio of 10:1, and then CNTs (0.6–6.0 wt %) were added to the mixture. The high viscosity PDMS/CNT paste was obtained by using a three-roll mill apparatus [23]. A double-roll machine was employed to produce the composite patches on a polymeric (polyimide, PI) substrate. When the viscous paste was placed in the nip between the rolls, all the material was transferred onto the roll with the substrate [24].

### 2.3. Surface Treatment of PDMS/CNT Composite Patches

The composite patch was treated with aqueous APTS solution (1 mM) overnight to provide surface compatibility. After complete drying, the composite patch was immersed in a chlorobutyric acid solution (CA, 30 mM) for 2 h. Subsequently, the solution containing the composite patch was mixed with *N*-hydroxysuccinimide (NHS, 0.1 M) and ethyl(dimethylaminopropyl) carbodiimide (EDC, 0.4 M) for bioconjugation. Finally, a mixture of beta-CD and an aqueous KOH solution (4 M, 20 mL) was introduced on top of the patch at 60–70 °C for 24 h, for the introduction of beta-CD onto the patch surface [25,26].

### 2.4. Instrumentation

Scanning electron microscopy (SEM) images were obtained on a Quanta 650 FEG (ThermoFisher Scientific, Hillsboro, OR, USA) to examine the morphology of the patches, depending on the CNT content. To observe the CNT dispersion condition in the PDMS matrix, the composite patches were quenched and broken in liquid nitrogen. The water contact angle (WCA) was acquired using a KRUSS drop shape analyzer (KRÜSS, Hamburg, Germany). FT-IR spectra were collected on a Perkin Elmer Spectrum One spectrometer (Perkin Elmer, Shelton, CT, USA). The four-wire resistance method was employed to measure the electrical conductivity of the composite patches using a Keithley 2400 Sourcemeter (Tektronix, Beaverton, OR, USA) and Keithley 487 picoammeter (Tektronix, Beaverton, OR, USA). All the electrical measurements were performed using a Keithley 2612B Sourcemeter (Tektronix, Beaverton, OR, USA) and a probe station MS TECH Model 4000 (MS Tech, Seoul, Korea) under ambient conditions.

## 3. Results and Discussion

### 3.1. Characterization and Surface Treatment of PDMS/CNT Composite Patch

The first important step of this study was the preparation of flexible PDMS/CNT composite patches (Figure S1). A range of CNT weight fraction was determined, as most physical and chemical properties of composite patches are strongly dependent on the CNT content. Taking into consideration the homogeneous dispersion of CNT into the PDMS matrix, the electrical conductivity, and the surface hydrophobicity of the patches, a CNT composition range of 0.6–6.0 wt % was selected. Below 0.6 wt %, it was impossible to achieve an appropriate and stable electrical conductivity due to the occurrence of a percolation threshold under 0.5 wt %. On the other hand, complete and uniform dispersion of CNT into the PDMS matrix became difficult above 6.0 wt % [27]. It was also feasible to produce flat and flexible PDMS/CNT composite patches within the CNT loading range by the process shown in Figure 1. In a previous article, where surface patterned PDMS/CNT patches were employed for a chemical sensor platform, 3–5 wt % of CNT was introduced for production of the patches [23].

**Figure 2:**
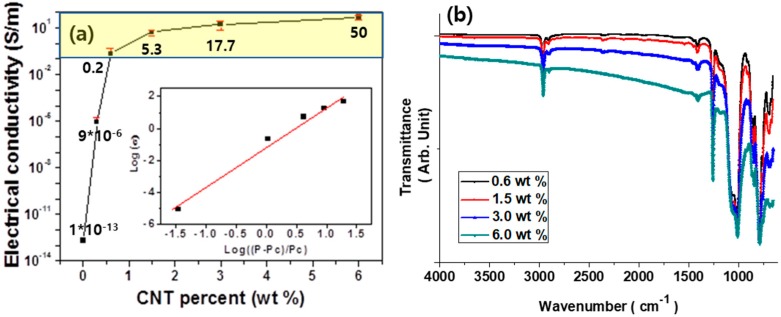
(**a**) Electrical conductivity and (**b**) FT-IR spectra of the PDMS/CNT composite patches as a function of the CNT composition.

Figure 2 presents the electrical conductivities and FT-IR spectra of the PDMS/CNT composite patches as a function of CNT composition. It is obvious that the electrical conductivity increased almost proportionally to the CNT weight fraction between 10^−1^ to 50 S/m (Figure 2a). This was because a percolation threshold phenomenon in the electrical conductivity occurred below 0.5 wt % for this composite system, as mentioned above [3]. This data supported that the CNT loading of 0.6–6.0 wt % was acceptable in this study. Figure 2b shows the FT-IR spectra of the PDMS/CNT composite patches. The characteristic peaks associated with the PDMS are apparent for all the spectra, which indicates that the surfaces of the composite patches were predominantly covered with the PDMS material. For example, the peaks for symmetric and asymmetric Si–O–Si stretching were observed at 800 and 1200 wavenumber, respectively. It was noteworthy that the baselines of the FT-IR spectra became more slanted as the CNT composition increased. This trend was associated with the plasma reflection, which is often observed in electrical conducting materials [2].

**Figure 3:**
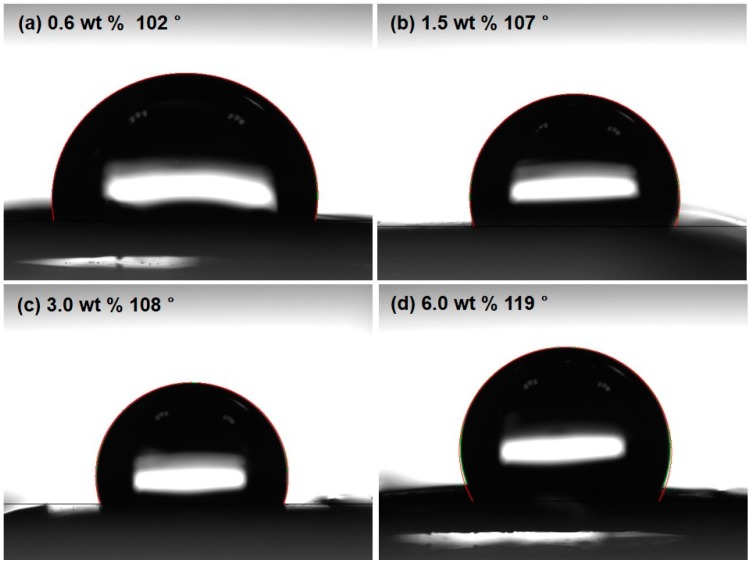
Contact angle measured on the surfaces of the PDMS/CNT composite patches as a function of CNT content.

Figure 3 presents the water contact angles of the PDMS/CNT composite patches measured on the surfaces. It was clear that the contact angle slightly increased from 102° to 119° with increasing CNT composition. This is attributed to the fact that the PDMS components were prevalent on the surfaces of the patches regardless of the CNT weight fraction. The addition of more CNT elevated the hydrophobicity of the patch surfaces.

**Figure 4:**
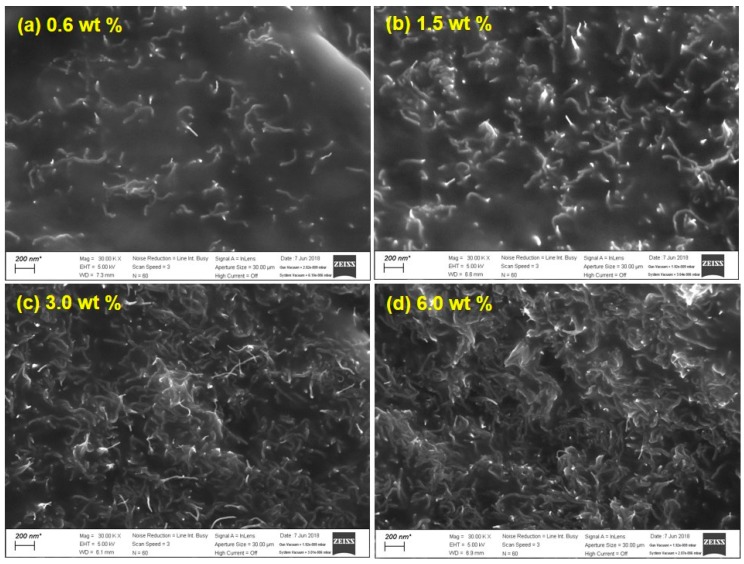
SEM images taken at the fractured surfaces of the PDMS/CNT composite patches.

In addition, it was also useful to examine the fractured surfaces of the PDMS/CNT composite patches in order to observe the CNT dispersion into the PDMS matrix. As this aspect was critical to finely control the electrical conductivity of the patches, SEM images of the fractured surfaces are displayed in Figure 4. The top-view image is presented in the supporting information (Figure S1). The overall features of the fractured surfaces seem equivalent, that is, CNT fillers are embedded in the polymer matrix. The population of CNT moieties increased with increasing CNT loading, therefore, more electrical paths were created inside the structure. From these observations, it is inferred that the PDMS/CNT composite patches can play the role as a robust and flexible platform for high performance chemical sensors. The mechanical property and stretchability/flexibility of the composite patch was demonstrated [3].

**Figure 5:**
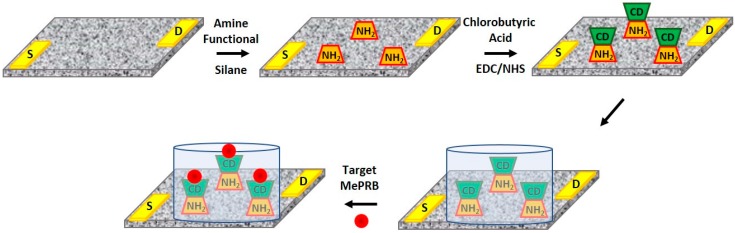
A schematic illustration of the surface treatment procedure to introduce beta-CD molecules as sensing media on the PDMS/CNT composite patches and sensor geometry, in order to measure sensing signals.

The produced composite patch as a flexible sensor platform was further modified at the surface to incorporate sensing media (beta-CD). Figure 5 shows a schematized process for this surface treatment. The electrodes were introduced by a simple lithographic technique and thermal deposition of Au metal. The PDMS/CNT patch was immersed in a silane coupling agent solution to provide functional groups on the surface for a successive bioconjugation step. In this experiment, a well-established bio-conjugation method—the EDC/NHS coupling reaction—was employed using chlorobutyric acid as an intermediate. Subsequently, the beta-CD molecules were attached to the patch surface via strong covalent bonding. The feasibility of this process was demonstrated in a previous article [3,25]. The successful introduction of the beta-CD molecules was confirmed by FT-IR spectroscopy (Figure S2). For this study, it was also important to introduce an equal amount of the sensing media, beta-CD molecules onto the patch surfaces. This was achieved by controlling the concentrations of the chemicals that were used, as the surface properties of the patches were found to be almost identical to those examined previously [26].

Sensor signals were obtained using a glass tube containing analyte solution. That is, the signal was recorded in the presence of the solvent. This geometry was more reliable, because signal noise could be minimized and the solvent in the glass tube could act as a buffer solution.

### 3.2. Sensing Signal Profile in Flexible Chemical Sensor

In this study, acquisition of a discernible sensing signal was a critical step to secure sensing performance. This was possible because the composite patch showed a sufficiently high electrical conductivity even at a low CNT loading. Sometimes, high conductivity led to an increase in noise level, therefore, compromise between conductivity and signal fidelity was substantially important.

**Figure 6:**
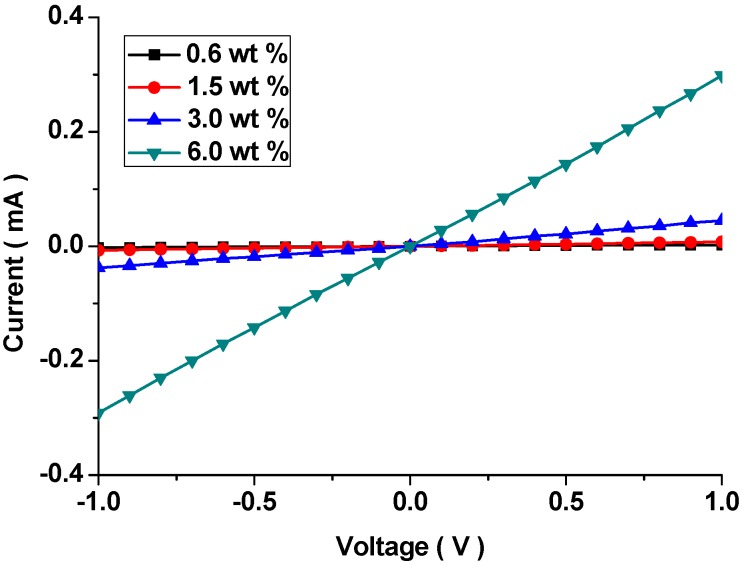
I–V curves of the surface modified PDMS/CNT composite patches as a function of CNT loading, showing the ohmic relation.

To this end, securing ohmic contact in the PDMS/CNT composite patches was indispensable. Figure 6 shows the I–V curves of the surface modified PDMS/CNT composite patches as a function of CNT loading.

For all cases, linear profiles were observed in the voltage range. As the weight fraction of CNT increased, the current also increased linearly. The current level was meaningful, because it determined the base current level during electrical sensing measurement.

Subsequently, sensor tests were performed. The first step was to monitor solvent effect on the sensor signal. This aspect was examined in detail in our previous articles [3]. Particularly, the sensor signal was found to be significantly dependent on the polarity of solvents. The signal variation increased slightly with increasing polarity of the solvent. In this work, a common solvent—ethanol—with a medium polarity was employed. As all the measurements were conducted under ethanol atmosphere, the effect of the solvent on sensor signal was identical for all PDMS/CNT composite patches (Figure S3).

**Figure 7:**
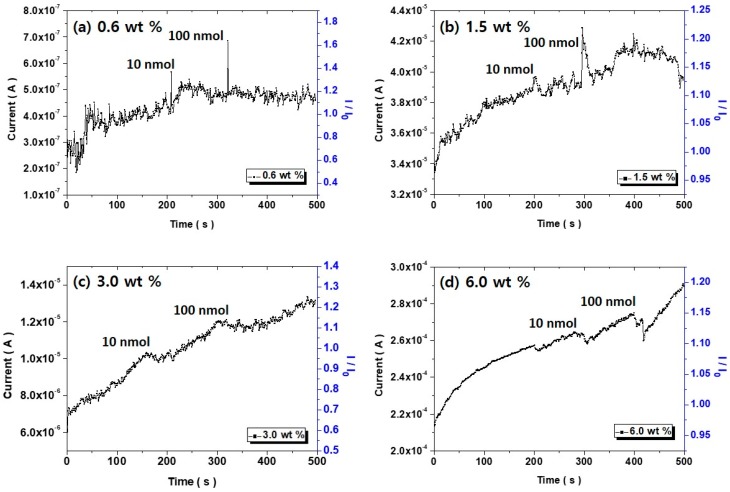
Sensor signal profiles of composite patches as a function of the CNT fraction, measured after the addition of varying amount of the target compound MePRB.

The sensing signal of flexible chemical sensors based on a surface treated PDMS/CNT composite patch was measured by changing the concentration of the model compound, MePRB from 10 to 100 nmol as shown in Figure 7. When the model compound MePRB was added to the sensor system, there were four types of interactions, which could contribute to the occurrence of signal peaks. These included patch-solvent, patch-model compound MePRB, CD-solvent, and CD-model compound MePRB. Supplementary experiment results showed that the patch-solvent and the CD-solvent interaction were negligible in terms of signal intensity (Figure S3). The patch-model compound MePRB interaction must be insignificant because a small amount of the model compound (10–100 nmol) was introduced to the sensor system during measurement. Therefore, the remarkable sensor signals in Figure 7 were strongly associated with the CD-model compound MePRB interaction. This was spontaneous and thermodynamically favorable because a host–guest complex could be generated between CD and MePRB, where the MePRB molecules could reside in the intrinsic pores of the CD molecules [28]. In this way, charge transport between CD and the model compound MePRB could be facilitated. This caused an increase in current values, leading to an uprise of signal peaks.

It was shown that the base currents increased gradually and continuously for all the experiments. There are two types of sensors, which are based on the change in conductance and resistance, respectively. The sensor system used in this study is based on the change in conductance, which is the ability for electric charge to flow in a certain path. The incorporation of external molecules such as solvent could induce regional movements of the CNT bundles. This might induce an effect of increasing the electrical paths inside PDMS/CNT composite patches. In addition, the charge transport through CNT network could be facilitated owing to the introduction of the analyte molecules, in this case, by formation of the host–guest complexes. These two factors might contribute to increase of the conductance of the PDMS/CNT composite patches.

**Figure 8:**
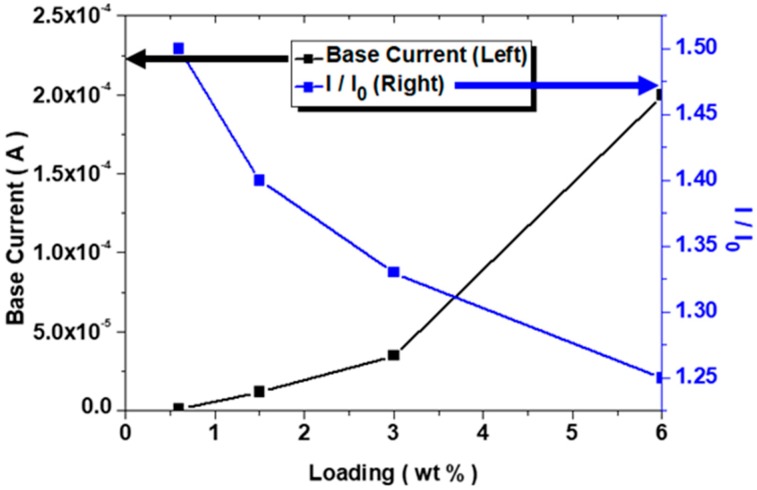
Variation of the base current and intensity of the sensor signal as a function of CNT loading.

In general, the signal peak intensity seemed to be proportional to the analyte concentration even if selectivity was also important. However, it was irrelevant to this study as only one target compound MePRB was incorporated. As the CNT content increased, the frequency of noise evolution decreased. Because electrical conductivity increased with an increasing CNT weight fraction, signal transduction was promoted due to increased conducting pathways in the composite patch. Therefore, more stable profiles could be recorded. In addition, the shape of the sensor signals became less sharp. This phenomenon was associated with the contribution of the charge transport from the CD-target compound MePRB interaction becoming less significant at high CNT loading, where the base current value was relatively high. Therefore, an antagonistic relationship between the base current and signal peak intensity was exhibited, as in Figure 8. For this layout, the base current was determined by the slope of ohmic plots in Figure 6. As the base current is not an absolute value, the current in Figure 7b seemed a bit higher than that in Figure 7c. Even if the values in blue curve were obtained from the measurements with 100 nmol solutions, the shape of the curve was independent of the concentration. For 1 nmol, the sensor signal was indistinctive (Figure S4).

Considering the data, it was possible to select a process window by optimizing dominant parameters such as electrical conductivity associated with the CNT content, base current, peak intensity (I/I_0_), and noise level for the performance improvement and effective use of flexible chemical sensors. Complementary experiments to demonstrate the sensing capability under bending and repetitive measurements is actively underway as a detailed study.

## 4. Conclusions

In this work, a close examination of sensing signal profiles of flexible chemical sensors, based on a surface engineered PDMS/CNT composite patch was performed using electrical measurements. The sharpness of the signal peaks was remarkable at low CNT loading, while the stability of the sensor signals improved with high CNT content. It was therefore feasible to select an optimized process window for flexible chemical sensors showing ultrahigh performance. This study is an exemplary demonstration for the improvement of sensor performances under several contradictory parameters. This article provides essential information for future research activities on ultrasensitive flexible chemical sensors based on PDMS/CNT composites.

## Supplementary Materials

The following are available online at http://www.mdpi.com/2073-4360/10/9/951/s1, Figure S1: A photograph and a top-view SEM image of the PDMS/CNT composite patch containing 3 wt % CNT, Figure S2: FT-IR spectra of PDMS/CNT composite patched as a function of CNT weight fraction obtained after each surface modification steps, Figure S3: Sensor signals obtained with PDMS/CNT composite patches after introduction of pure solvent to the sensor system, Figure S4: Sensor signals obtained with PDMS/CNT composite patches after introduction of 1 nmol solution to the sensor system.
