# Supplementary material for: Study on the Sensing Signal Profiles for Determination of Process Window of Flexible Sensors Based on Surface Treated PDMS/CNT Composite Patches
Source: Polymers (Basel). 2018 Aug 27;10(9):951. doi: 10.3390/polym10090951 (PMC6403834; doi:10.3390/polym10090951)
Supplement: Supplementary file 1 [file polymers-10-00951-s001.pdf]

Supplementary Materials for

Study on the Sensing Signal Profiles for Determination of Process Window of Flexible Sensors Based on Surface Treated PDMS/CNT Composite Patches

Joonwon Bae, Yunjung Hwang, Seon Joo Park,
Ji-Hwan Ha, Hye Jun Kim, Ayeon Jang, Jaieun An,
Chang-Soo Lee,* Sung-Hoon Park*

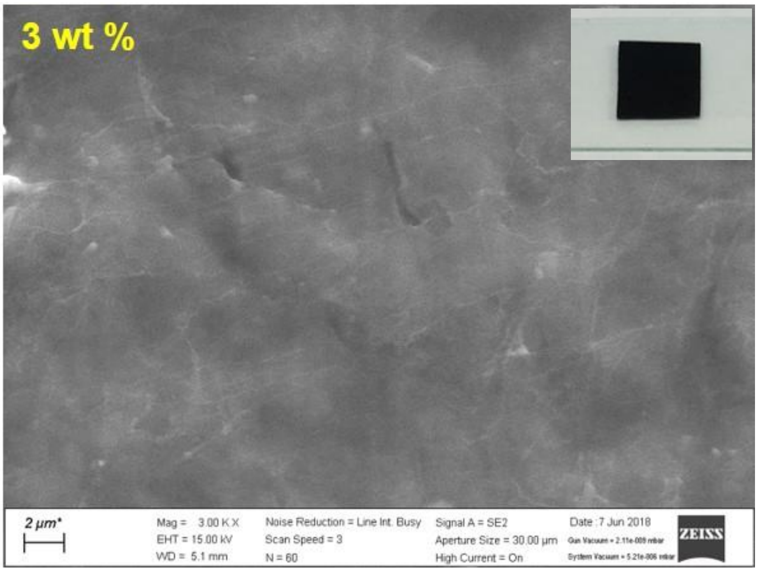

Figure S1. A photograph and a top-view SEM image of the PDMS/CNT composite patch containing 3 wt % CNT.

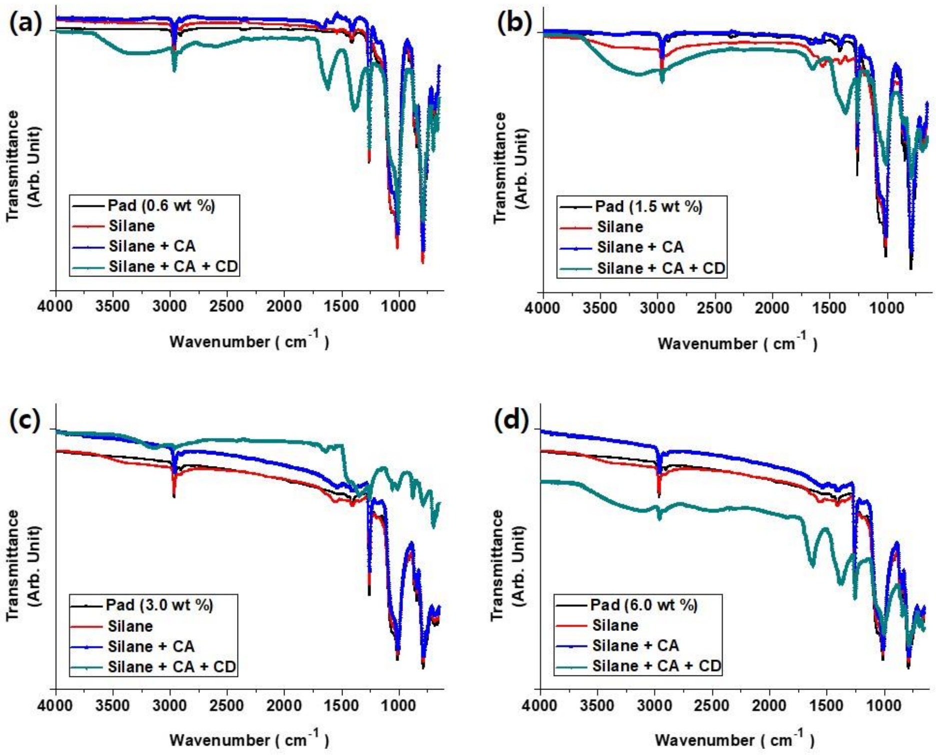

Figure S2. FT-IR spectra of PDMS/CNT composite patched as a function of CNT weight fraction obtained after each surface modification steps.

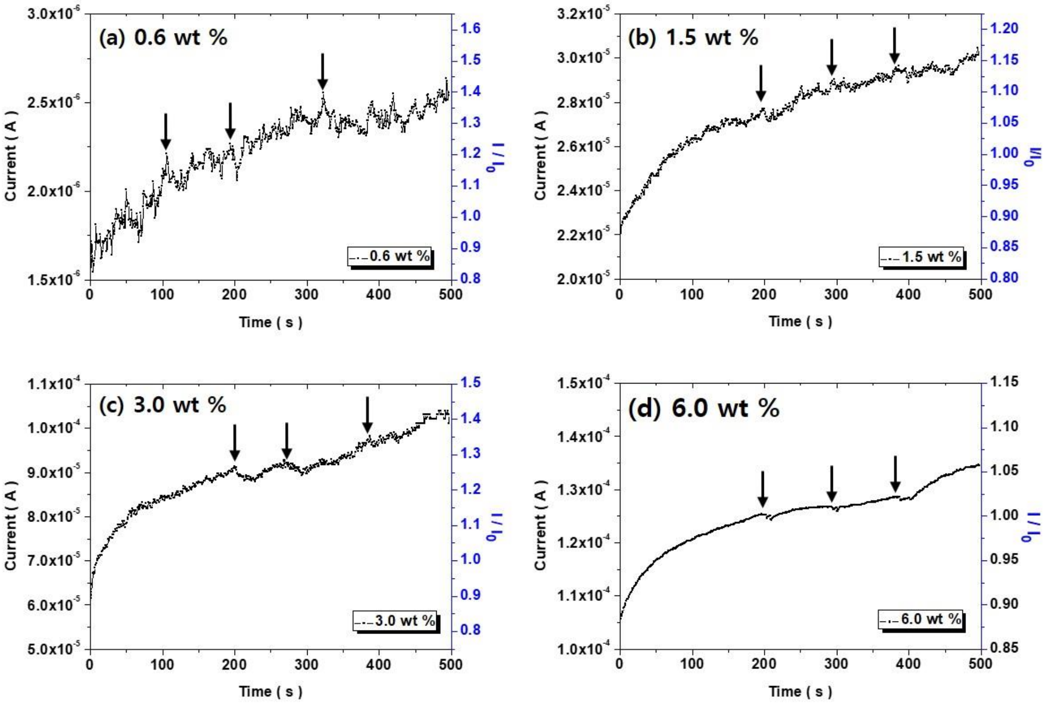

Figure S3. Sensor signals obtained with PDMS/CNT composite patches after introduction of pure solvent to the sensor system.

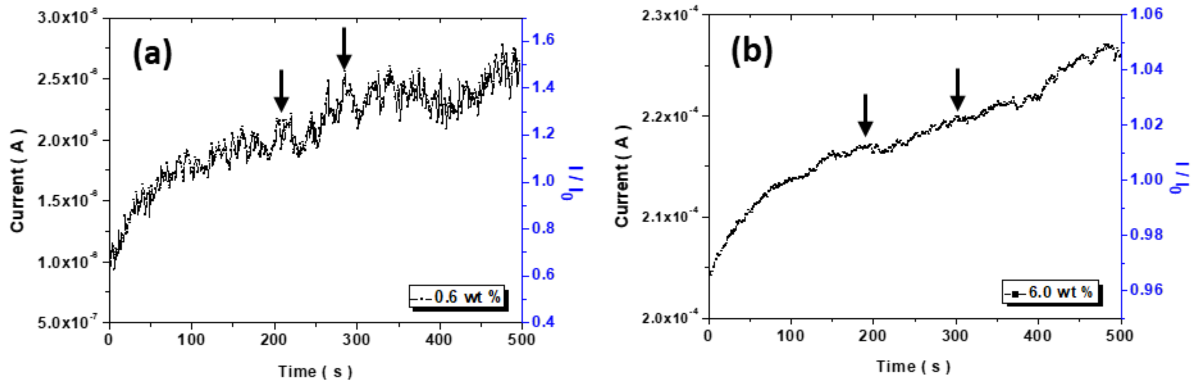

Figure S4. Sensor signals obtained with PDMS/CNT composite patches after introduction of 1 nmol solution to the sensor system.
